# Effect of classical music on growth performance, stress level, antioxidant index, immune function and meat quality in broilers at different stocking densities

**DOI:** 10.3389/fvets.2023.1227654

**Published:** 2023-08-03

**Authors:** Xinlei Gao, Jiangang Gong, Bowen Yang, Yanci Liu, Hongjian Xu, Yanshuang Hao, Jialin Jing, Zhihua Feng, Lihua Li

**Affiliations:** ^1^College of Animal Science and Technology, Hebei Agricultural University, Baoding, China; ^2^College of Food Science and Technology, Hebei Agricultural University, Baoding, China; ^3^Baoding Vocational and Technical College, Baoding, China; ^4^Hebei Jiuxing Agriculture and Animal Husbandry Development Co., Ltd., Baoding, China; ^5^College of Mechatronical and Electrical Engineering, Hebei Agricultural University, Baoding, China

**Keywords:** stocking density, classical music, broilers, growth performance, stress, antioxidation, immune function, meat quality

## Abstract

High-stocking density is one of the factors that can easily cause oxidative stress and inflammatory reaction of broilers. Currently, music therapy has been proposed to help animals relieve stress to some extent. However, it is still unclear whether classical music can alleviate stress in broilers at high stocking densities. Hence, this study aimed to investigate the effects of classical music on growth performance, stress level, antioxidant index, immune function and meat quality of broilers under different stocking densities. A total of 540 one-day-old broilers with similar body weight were randomly divided into 6 treatment groups, with 6 replicates per group, which included two feeding environments (with/without classical music) and three stocking densities (15.5, 17.9, and 20.3 birds/m^2^), thereby making a 2 × 3 factorial arrangement. The results showed as follows: increasing stocking density decreased the average daily feed intake and average daily gain (ADG), increased feed-to-gain ratio (F/G) and mortality of broilers. Moreover, increased density resulted in an increase in serum corticosterone (CORT) and adrenocorticotropic hormone (ACTH) levels. Increasing stocking density decreased spleen and bursal indices, serum immunoglobulin A (IgA), immunoglobulin G (IgG), and immunoglobulin M (IgM) levels. Increasing stocking density elevated serum malondialdehyde (MDA) and decreased catalase (CAT), superoxide dismutase (SOD) and glutathione peroxidase (GSH-PX) activities. Increasing stocking density decreased serum total protein (TP) levels and increased total cholesterol (TC) and glucose (GLU) levels. Additionally, increasing stocking density decreased the cooking liss of pectoralis and increased the L*_24h_ value of pectoralis. Meanwhile, playing classical music for broilers increased their ADG and decreased F/G, and decreased serum CORT, ACTH, GLU content. In addition, the bursa of Fabricius index, serum IgA and IgG contents as well as the a*_24h_ value of pectoralis was increased under the music therapy. In conclusion, high-stocking density (20.3 birds/m^2^) harmed the growth performance and health of broilers, and the classical music stimulus ameliorated the negative effects to some extent.

## 1. Introduction

In the modern poultry industry, high density is a common phenomenon in broiler rearing, which enables producers to reduce rearing costs and gain more profit. Regarding the breeding standard of broiler stocking density, it varies widely among different countries. For example, the welfare guidelines of the National Chicken Council advocate an ideal rearing density of 41.5 kg/m^2^ for broilers over 2 kg (1), the stocking densities in Netherlands, the United Kingdom, and Switzerland are 45–54 kg/m^2^, 40 kg/m^2^, and 30–36 kg/m^2^, respectively (2). It is well known that high-density stocking is a major contributor to broiler stress. Intensive stocking not only increases corticosterone levels in broilers to induce physiological stress (3) but also decreases average daily feed intake and average daily gain and increases feed-to-gain ratio in broilers (4). Li et al. (5) indicated that serum immunoglobulins (immunoglobulin G and immunoglobulin A) decreased significantly in broilers as stocking density increased from 15 to 18 birds/m^2^, which also led to an imbalance in serum antioxidant levels (decreased superoxide dismutase and glutathione peroxidase activities and increased malondialdehyde levels). Simsek et al. (6) also indicated that when broiler stocking density reached 47.3 kg/m^2^ may lead to oxidative stress and a decrease in bursal weight in broilers. Therefore, how to mitigate some of the adverse effects of increased stocking density on broilers has become an urgent issue in large-scale broiler breeding.

In humans, music is widely used to enhance well-being, reduce stress, and distract patients from unpleasant symptoms (7). Music therapy is now being used to improve the environment of farm animals, which is considered an effective method to improve animal welfare. Playing music can not only cover up the background noise in the house environment, but also reduce anxiety, stress, and aggressive behavior by providing auditory enrichment. For example, cows exposed to classical music showed better performance in terms of milking time, milking speed, cortisol hormone and behavioral parameters (reduction in stereotyped behavior, etc.) (8). Listening to classical music has a calming effect on pet dogs in the stressful environment of veterinary hospitals (9). Short-term classical music stimulation increased activity, tail-dragging and playful behavior in fattening pigs, while long-term music stimulation increased serum interleukin-2, interferon-γ and immunoglobulin levels and decreased serum interleukin-4 levels (10). Early studies suggested that music altered hypothalamic–pituitary–adrenal axis activity by modulating the hypothalamus (11, 12), inducing an increase in dopamine levels in the brains of chicks and hypertensive rats, and inhibiting sympathetic activity (13, 14). The well-developed cochlea of birds has been reported to guide the recognition of audio and thus can process relative tones as in the primary auditory cortex of most mammals (15). African grey parrots and sulfur-crested cockatoos spontaneously move in sync with the music (head bobbing and foot lifting) (16), suggesting that birds also have the ability to perceive external rhythms like mammals. Furthermore, according to our previous study, classical music was found to have better effects on broiler health compared to popular and ethnic music (17).

Applying music to the production of animal farming is simple and easy. However, to the best of our knowledge, no studies have been reported on the effects of classical music on broilers, not to mention at high densities. Therefore, in our study, the effects of classical music on growth performance, stress level, antioxidant index, immune function and meat quality of broiler chickens at different densities were investigated.

## 2. Materials and methods

### 2.1. Birds, experimental design, and management

Broilers in this study were purchased from Jiuxing Hatchery (Jiuxing Agricultural and Livestock Development Co., Ltd., Baoding, China). A total of 540 one-day-old Cobb broilers with average body weight of (44.66 ± 0.05 g) were randomly divided into six treatment groups with six replicates per group. The six treatment groups included two feeding environments (with/without classical music) and three stocking densities (15.5, 17.9, and 20.3 birds/m^2^). The number of chickens per replicate for each density was 13, 15 and 17, respectively. The experiment lasted for 42 d. Classical music selected from Indian Raga included *Raga khamaj*, *Raga yaman*, *Raga Gunkali*, *Raga Durga*, *Raga jayajavanti*, *Raga bhupali*, and *Raga darbari*. The tracks used in this study are searchable on Himalayan Audio Platform.^1^

The experiment was conducted from June 21 to August 1, 2022. Two environmental control rooms (L × W × H = 10 m × 5 m × 3 m) with the same feeding level and adjacent to each other were selected. The broilers without music in the house were taken as the control group, and broilers with music in the other coop were used as the music treatment group. Broilers in the experimental group began to play music from 1 day of age. There is no sound interference between the two houses. The chicken coop is a three-layer superimposed cage with a single cage area of 0.84 m^2^ and semi-mechanical ventilation. The music playing cycle is 3 h each from 5:00 to 8:00, 9:00 to 12:00, 16:00 to 19:00, and 20:00 to 23:00 every day. The digital noise meter was used to measure the volume in the house. Two smart loudspeakers with the same specifications were placed 2 m above the music treatment house. The speakers were connected *via* an audio cable. The two speakers played music at the same time so that the music can evenly cover the whole music treatment house. The volume was kept constant during the playing process, and the music decibel was set at 65–75 dB. During the experiment, all birds were allowed to feed and drink freely. The temperature remained at 34°C for the first week, then gradually decreased to 24°C at a rate of 3–4°C per week, and remained unchanged thereafter. From 14 to 35 d of age, broilers were kept in light for 20 h and dark for 4 h, and the remaining days were kept in light for 24 h. The average relative humidity in the chicken house was kept between 60% and 70%, and the chickens were fed according to the routine feeding management procedures and immunization procedures. The rations were prepared using a corn-soybean meal basal diet, referring to the Chinese Chicken Feeding Standard (NY/T3645-2020) and the National Research Council (18). The composition and nutritional levels of the basal diet are shown in Table 1.

**Table 1:** Composition and calculation analysis of the basal diets (% dry basis).

Items	Content (%)		
	1–14 d of age	15–35 d of age	36–42 d of age
Ingredients			
Corn	53.20	55.80	58.80
Soybean meal (43%CP)	25.90	20.10	15.30
Wheat flour	8.00	10.00	10.00
Corn gluten meal (60%CP)	2.90	2.30	3.00
Peanut meal	2.70	2.60	3.10
Feather meal	1.00	1.50	2.00
Soya-bean oil	1.48	3.10	3.50
CaHPO4	1.40	1.20	0.90
Limestone	1.20	1.16	1.20
L-methionine	0.22	0.24	0.20
Premix^a^	2.00	2.00	2.00
Total	100	100	100
Nutrient levels^b^			
ME/(MJ/kg)	12.26	12.89	13.18
CP	21.20	19.20	18.50
Lys	1.43	1.27	1.20
Met+Cys	0.94	0.91	0.88
Thr	0.94	0.88	0.85
Trp	0.22	0.19	0.17
Ca	0.90	0.83	0.80
TP	0.52	0.44	0.40

a Premix is a diet containing: Vitamin A 12,000 IU; Vitamin B_1_ 3 mg; Vitamin B_2_ 9 mg; Vitamin B_6_ 4.5 mg; Vitamin B_12_ 0.03 mg; Vitamin D_3_ 4,500 IU; Vitamin E 36,000 mg; Vitamin K 36,000 mg; Vitamin H 0.18 mg; D-Calcium-Pantothenate 13.5 mg; folic acid 1.8 mg; Nicotinic acid 45 mg; Fe 110 mg; Cu 16 mg; Mn 120 mg; Zn 110 mg; Se 0.35 mg; Iodine 1.5 mg.

b Nutrient levels were all calculated values.

### 2.2. Growth performance

The total body weight and total feed intake of 42-day-old chickens were recorded for each replicate, and the daily gain and feed conversion ratio were calculated. The overall mortality rate for each replicate was considered as the total mortality rate during the experiment. The average daily feed intake (ADFI), average daily gain (ADG), feed-to-gain ratio (F/G), and mortality rate of broilers were calculated at the end of the experiment.

### 2.3. Serum stress, antioxidant, and biochemical indexes

The day the experiment ended (42 d), samples were selected from two average-weight broilers in each replicate after 12 h of fasting. Blood samples were collected from wing veins in 10 mL non-anticoagulated vacuum tubes, placed at room temperature, centrifuged at 3,000 r/min for 10 min to prepare serum, and stored at −20°C. The concentrations of serum corticosterone (CORT, NO. HY-10063) and adrenocorticotropin (ACTH, NO. HY-10175) were determined by the enzyme-free method, and creatine kinase (CK, N-acetylcysteine method) was determined by the colorimetric method. The kit was purchased from the Beijing Huaying Institute of Biotechnology (Beijing, China). Analysis of serum total antioxidant capacity (T-AOC, NO. A015-2-1), catalase (CAT, NO. A007-1-1), superoxide dismutase (SOD, NO. A001-3-2), glutathione peroxidase (GSH-PX, NO. A005-1-2) activity, and malondialdehyde (MDA, NO. A003-1-2) content, serum biochemical indices total protein (TP, NO. A045-2-2), albumin (ALB, NO. A028-2-1), total cholesterol (TC, NO. A112-2-1) and glucose (GLU, NO. F006-1-1) content were carried out according to the instructions of the kit Nanjing Jiancheng Bioengineering Institute (Nanjing, Jiangsu, P.R. China).

### 2.4. Immunologic function

Selected broilers were dislocated and executed by neck dislocation after blood collection, and the thymus, spleen and bursa were quickly removed for weighing and calculation of immune organ index after confirmation of death.

The contents of immunoglobulin A (IgA, NO. H108-1-2), immunoglobulin M (IgM, NO. H109-1-2), and immunoglobulin G (IgG, NO. H106-1-2) in serum were detected with chicken specific enzyme-linked immunosorbent assay kit purchased from Nanjing Jiancheng Institute of Bioengineering(Nanjing, Jiangsu, P.R. China).

### 2.5. Meat quality determination

The selected broilers were slaughtered and then uniformly stripped of the left pectoral muscle for 24 h. The lightness (L*), redness (a*), and yellowness (b*) of the pectoral muscle were determined using a CR-10 colorimeter (Tokyo, Japan) 24 h after stripping the left pectoral muscle of each slaughtered broiler. Cooking loss and shear force were determined by referring to Cai et al. (19).

### 2.6. Statistical analysis

The experimental data were expressed as “mean ± standard deviation,” and the general linear model (GLM) program in SPSS 26.0 statistical software was used for two-factor ANOVA. The main effects of the model included classical music and stocking density and their interaction effects. When the interaction effects were significantly different, Duncan’s method was used to compare the significance of the difference between the mean values of each group. *p* < 0.05 indicates a significant difference, while 0.05 ≤ *p* < 0.10 indicates a trend of difference. The mortality of broiler was analyzed by the Chi-square test.

## 3. Results

### 3.1. Growth performance

As shown in Table 2, different stocking densities had significant effects on ADFI, ADG, F/G, and mortality of broilers (*p* < 0.05). The ADFI in the 15.5 birds/m^2^ and 17.9 birds/m^2^ groups was higher than that in the 20.3 birds/m^2^ group (*p* < 0.05). Increasing stocking density decreased the ADG of broilers (*p* < 0.05). The F/G of the 15.5 birds/m^2^ group was lower than that of the 17.9 birds/m^2^ group and 20.3 birds/m^2^ group (*p* < 0.05). With the increase in stocking density, the mortality of broilers increased (*p* < 0.05). Playing classical music increased ADG and decreased the F/G of broilers (*p* < 0.05). After classical music intervention, broilers F/G was significantly lower in the 17.9 and 20.3 birds/m^2^ groups than in the group without music intervention (*p* < 0.05). The effect of interaction on broilers F/G was observed, where playing classical music significantly mitigated the increase in broilers F/G caused by increased stocking density at 17.9 and 20.3 birds/m^2^ (*p* < 0.05).

**Table 2:** Effect of classical music and stocking density on growth performance of broilers.

Items		ADFI/g	ADG/g	F/G	Mortality (%)
Stocking density (birds/m^2^)	Classical music				
15.5	+	104.76 ± 3.58	58.36 ± 1.90	1.80 ± 0.03^d^	1.28 ± 3.14
	−	104.66 ± 2.38	57.57 ± 1.51	1.82 ± 0.03^cd^	1.28 ± 3.14
17.9	+	103.40 ± 3.19	55.99 ± 1.04	1.85 ± 0.03^c^	2.22 ± 3.44
	−	104.68 ± 1.99	54.52 ± 0.87	1.92 ± 0.03^b^	6.67 ± 4.21
20.3	+	99.19 ± 1.46	52.31 ± 1.30	1.90 ± 0.03^b^	5.88 ± 5.26
	−	99.51 ± 3.50	50.56 ± 1.88	1.97 ± 0.02^a^	8.82 ± 3.22
Main factors					
Classical music	+	102.45 ± 3.65	55.55 ± 2.92	1.85 ± 0.05	3.13 ± 4.32
	−	102.95 ± 3.56	54.22 ± 3.26	1.90 ± 0.07	5.59 ± 4.67
Stocking density (birds/m^2^)	15.5	104.71 ± 2.90^a^	57.97 ± 1.68^a^	1.80 ± 0.03^b^	1.28 ± 2.99^c^
	17.9	104.04 ± 2.60^a^	55.25 ± 1.19^b^	1.88 ± 0.05^a^	4.44 ± 4.34^b^
	20.3	99.35 ± 2.56^b^	51.44 ± 1.79^c^	1.93 ± 0.04^a^	7.35 ± 4.43^a^
*p*-value					
Classical music		0.166	0.010	<0.001	0.691
Stocking density		<0.001	<0.001	<0.001	<0.001
Interaction		0.595	0.720	0.046	

^a–d^Means within a row column with no common superscripts differ significantly (*p* < 0.05). ADFI, average daily feed intake; ADG, average daily gain; F/G, feed-to-weight ratio; Interaction, indicates the interaction effect of classical music and stocking density.

### 3.2. Serum stress indices

Serum CORT and ACTH of broilers were affected by different stocking densities (*p* < 0.05). With the increase in stocking density, serum CORT and ACTH contents of broilers were decreased (*p* < 0.05). The serum CK content of broilers had an increasing trend (*p* = 0.067) with the increase in stocking density. Playing classical music increased serum CORT and ACTH contents of broilers (*p* < 0.05). There was an interaction between classical music and stocking density on serum CORT and ACTH levels in broilers (*p* < 0.05). Serum CORT and ACTH levels in the 17.9 birds/m^2^ music group decreased to the same level as in the 15.5 birds/m^2^ group (*P*>0.05). Serum CORT and ACTH levels in the 20.3 birds/m^2^ music group decreased to the same level as in the 17.9 birds/m^2^ no music group (*p*>0.05). In addition, the serum CORT and ACTH levels of broilers in the music group at the same stocking density were significantly lower (*p* < 0.05), indicating that classical music can reduce the increase in stress levels in broilers caused by increased stocking density (Table 3).

**Table 3:** Effects of classical music and stocking density on serum stress indices of broilers.

Items		CORT (ng/mL)	ACTH (pg/mL)	CK (U/L)
Stocking density (birds/m^2^)	Classical music			
15.5	+	4.58 ± 0.41^c^	26.64 ± 2.31^c^	2877.02 ± 275.60
	−	4.63 ± 0.50^c^	29.54 ± 2.11^c^	2908.15 ± 213.07
17.9	+	5.13 ± 0.45^c^	27.71 ± 2.53^c^	3047.47 ± 269.70
	−	6.10 ± 0.67^b^	36.36 ± 3.90^b^	3049.21 ± 300.14
20.3	+	6.18 ± 0.59^b^	33.29 ± 2.33^b^	3097.50 ± 233.03
	−	7.37 ± 0.63^a^	40.03 ± 2.36^a^	3213.58 ± 299.41
Main factors				
Classical music	+	5.29 ± 0.82	29.22 ± 3.75	3007.33 ± 261.14
	−	6.03 ± 1.28	35.31 ± 5.24	3056.98 ± 287.59
Stocking density (birds/m^2^)	15.5	4.60 ± 0.44^c^	28.09 ± 2.60^c^	2892.58 ± 235.42
	17.9	5.61 ± 0.74^b^	32.04 ± 5.50^b^	3048.34 ± 269.36
	20.3	6.77 ± 0.85^a^	36.66 ± 4.17^a^	3155.54 ± 262.88
*p-*value				
Classical music		<0.001	<0.001	0.580
Stocking density		<0.001	<0.001	0.067
Interaction		0.040	0.038	0.862

^a–c^Means within a row column with no common superscripts differ significantly (*p* < 0.05). CORT, corticosterone; ACTH, adrenocorticotropic hormone; CK, creatine kinase; interaction: indicates the interaction effect of classical music and stocking density.

### 3.3. Serum antioxidant capacity

The activities of CAT, SOD, MDA and GSH-PX in broiler serum were affected by different stocking densities (*p* < 0.05; Table 4). Serum CAT activity of broilers in 15.5 birds/m^2^ and 17.9 birds/m^2^ groups was higher than that in the 20.3 birds/m^2^ group (*p* < 0.05). The serum SOD activity of the 15.5 birds/m^2^ group was higher than that of the 17.9 birds/m^2^ group and 20.3 birds/m^2^ group (*p* < 0.05). With the increase in stocking density, the serum MDA content of broilers was increased (*p* < 0.05). Increasing density resulted in a decrease in serum GSH-PX activity of broilers (*p* < 0.05). Playing classical music decreased the serum MDA content of broilers (*p* < 0.05), and tended to increase the activities of serum SOD and GSH-PX (*p* = 0.079, *p* = 0.061). There was no interaction between classical music and stocking density on the serum antioxidant capacity of broilers (*p* > 0.05).

**Table 4:** Effects of classical music and stocking density on serum immune function of broilers.

Items		Thymus (g/kg)	Spleen (g/kg)	Bursa of Fabricius (g/kg)	IgA (g/L)	IgM (g/L)	IgG (g/L)
Stocking density (birds/m^2^)	Classical music						
15.5	+	1.90 ± 0.11	0.56 ± 0.15	0.49 ± 0.10	2.74 ± 0.11	1.21 ± 0.11	5.07 ± 0.13^a^
	−	1.83 ± 0.13	0.50 ± 0.11	0.42 ± 0.04	2.70 ± 0.10	1.25 ± 0.06	5.14 ± 0.13^a^
17.9	+	1.85 ± 0.35	0.52 ± 0.08	0.50 ± 0.04	2.39 ± 0.16	1.19 ± 0.06	4.92 ± 0.21^a^
	−	1.73 ± 0.17	0.43 ± 0.05	0.46 ± 0.07	2.35 ± 0.19	1.17 ± 0.07	4.44 ± 0.30^b^
20.3	+	1.74 ± 0.21	0.44 ± 0.06	0.40 ± 0.07	2.45 ± 0.18	1.16 ± 0.05	4.11 ± 0.21^c^
	−	1.62 ± 0.21	0.40 ± 0.06	0.36 ± 0.07	2.10 ± 0.08	1.13 ± 0.08	4.02 ± 0.10^c^
Main factors							
Classical music	+	1.83 ± 0.21	0.51 ± 0.11	0.46 ± 0.08	2.54 ± 0.22	1.19 ± 0.08	4.81 ± 0.38
	−	1.73 ± 0.19	0.44 ± 0.09	0.42 ± 0.07	2.39 ± 0.29	1.18 ± 0.08	4.66 ± 0.49
Stocking density (birds/m^2^)	15.5	1.86 ± 0.12	0.53 ± 0.13^a^	0.46 ± 0.08^a^	2.72 ± 0.10^a^	1.23 ± 0.09^a^	5.10 ± 0.13^a^
	17.9	1.79 ± 0.27	0.47 ± 0.08^ab^	0.48 ± 0.06^a^	2.37 ± 0.17^b^	1.18 ± 0.06^ab^	4.68 ± 0.35^b^
	20.3	1.68 ± 0.21	0.42 ± 0.06^b^	0.38 ± 0.07^b^	2.20 ± 0.20^c^	1.14 ± 0.06^b^	4.07 ± 0.16^c^
*p-*value							
Classical music		0.162	0.050	0.046	0.023	0.820	0.014
Stocking density		0.163	0.032	0.003	<0.001	0.020	<0.001
Interaction		0.950	0.756	0.820	0.078	0.554	0.005

^a–c^Means within a row column with no common superscripts differ significantly (*p* < 0.05). IgA, ImmunoglobulinA; IgM, ImmunoglobulinM; IgG, ImmunoglobulinG; Interaction, indicates the interaction effect of classical music and stocking density.

### 3.4. Serum biochemical indices

As shown in Table 5, serum TP, TC and GLU levels in broiler were influenced by different stocking densities (*p* < 0.05). The serum TP content in the 15.5 birds/m^2^ group was higher than that in the 20.3 birds/m^2^ group (*p* < 0.05). Increasing stocking density increased serum TC levels of broilers (*p* < 0.05). The content of serum GLU in the 15.5 birds/m^2^ group was lower than that in the 20.3 birds/m^2^ group (*p* < 0.05). Serum ALB tended to decrease (*p* = 0.078) by increasing stocking density. Playing classical music decreased the serum GLU content of broilers (*p* < 0.05), and tended to decrease the serum TC content (*p* = 0.083). There was no interaction effect between classical music and stocking density on the serum biochemical indices of broilers (*p* > 0.05).

**Table 5:** Effects of classical music and stocking density on serum antioxidant capacity of broilers.

Items		T-AOC (nmol/L)	CAT (U/mL)	SOD (U/mL)	MDA (nmol/mL)	GSH-PX (U/mL)
Stocking density (birds/m^2^)	Classical music					
15.5	+	0.91 ± 0.03	10.83 ± 0.87	63.73 ± 5.81	4.14 ± 0.29	945.72 ± 43.59
	−	0.92 ± 0.03	10.45 ± 0.73	60.24 ± 3.16	4.86 ± 0.55	935.48 ± 46.56
17.9	+	0.90 ± 0.04	10.78 ± 0.72	55.71 ± 2.55	4.69 ± 0.39	913.75 ± 51.20
	−	0.90 ± 0.04	10.46 ± 0.55	59.24 ± 3.16	5.41 ± 0.49	811.23 ± 48.21
20.3	+	0.89 ± 0.03	9.68 ± 0.52	56.22 ± 3.57	5.09 ± 0.47	861.23 ± 48.21
	−	0.89 ± 0.03	9.22 ± 0.73	54.69 ± 4.41	5.63 ± 0.41	816.92 ± 39.96
Main factors						
Classical music	+	0.90 ± 0.03	10.43 ± 0.87	59.40 ± 5.07	4.80 ± 0.68	900.54 ± 61.55
	−	0.90 ± 0.03	10.04 ± 0.87	57.05 ± 4.27	5.14 ± 0.56	871.21 ± 65.76
Stocking density (birds/m^2^)	15.5	0.92 ± 0.03	10.64 ± 0.79^a^	61.98 ± 4.82^a^	4.42 ± 0.43^c^	940.60 ± 43.33^a^
	17.9	0.90 ± 0.03	10.62 ± 0.64^a^	57.49 ± 3.33^b^	4.98 ± 0.51^b^	887.49 ± 84.77^b^
	20.3	0.89 ± 0.03	9.45 ± 0.65^b^	55.20 ± 3.47^b^	5.52 ± 0.44^a^	829.53 ± 40.37^c^
*p-*value						
Classical music		0.917	0.103	0.079	0.031	0.061
Stocking density		0.124	<0.001	0.001	<0.001	<0.001
Interaction		0.949	0.969	0.737	0.606	0.516

^a–c^Means within a row column with no common superscripts differ significantly (*p* < 0.05). T-AOC, total antioxidant capacity; CAT, catalase activity; SOD, superoxide dismutase; MDA, malondialdehyde; GSH-Px, glutathione peroxidase; Interaction, indicates the interaction effect of classical music and stocking density.

### 3.5. Immune function

The indicators related to immune function were listed in Table 6, spleen and bursal indices of broilers were affected by different stocking densities (*p* < 0.05). The relative weight of the spleen and bursa of Fabricius in the 15.5 birds/m^2^ group was lower than that in the 20.3 birds/m^2^ group (*p* < 0.05). Playing classical music increased the relative weight of the bursa of Fabryssa (*p* < 0.05), and tended to increase the relative weight of the spleen (*p* = 0.050). There was no interaction effect between playing classical music and stocking density on the immune organ index of broilers (*p* > 0.05). The contents of IgA, IgM, and IgG in the serum of broilers were affected by different stocking densities (*p* < 0.05). Increasing stocking density decreased the serum IgA and IgG contents of broilers (*p* < 0.05). The serum IgM content of broilers in the 15.5 birds/m^2^ group was higher than that in the 20.3 birds/m^2^ group (*p* < 0.05). Playing classical music increased the serum IgA and IgG contents of broilers (*p* < 0.05). An interaction effect was observed in the serum IgM levels of broilers (*p* < 0.05). After playing classical music, the IgM content of the 17.9 birds/m^2^ group recovered to the same level as that of the 15.5 birds/m^2^ group (*p* > 0.05). It showed that classical music could improve the decrease of serum IgM level caused by the increase of stocking density (*p* < 0.05).

**Table 6:** Effect of classical music and stocking density on serum biochemical indexes of broilers.

Items		TP (g/L)	ALB (g/L)	TC (mmol/L)	GLU (mmol/L)
Stocking density (birds/m^2^)	Classical music				
15.5	+	48.44 ± 2.96	14.89 ± 1.05	1.97 ± 0.14	10.13 ± 0.93
	−	48.77 ± 2.36	14.68 ± 1.23	2.06 ± 0.12	11.51 ± 1.12
17.9	+	47.19 ± 2.23	13.98 ± 1.06	2.22 ± 0.11	10.92 ± 0.91
	−	46.91 ± 1.99	13.66 ± 1.18	2.29 ± 0.23	12.29 ± 1.25
20.3	+	46.11 ± 2.31	13.57 ± 0.90	2.56 ± 0.19	12.01 ± 1.61
	−	44.75 ± 3.31	13.26 ± 1.29	2.68 ± 0.11	12.71 ± 1.28
Main factors					
Classical music	+	47.25 ± 2.59	14.15 ± 1.10	2.25 ± 0.28	11.02 ± 1.37
	−	46.81 ± 2.29	13.86 ± 1.43	2.34 ± 0.30	12.17 ± 1.27
Stocking density (birds/m^2^)	15.5	48.61 ± 2.56^a^	14.78 ± 1.09^a^	2.04 ± 0.10^c^	10.82 ± 1.21^b^
	17.9	47.05 ± 2.02^ab^	13.82 ± 1.08^b^	2.25 ± 0.18^b^	11.60 ± 1.26^ab^
	20.3	45.43 ± 2.85^b^	13.41 ± 1.07^b^	2.62 ± 0.16^a^	12.36 ± 1.43^a^
*p-*value					
Classical music		0.615	0.459	0.083	0.008
Stocking density		0.019	0.017	<0.001	0.015
Interaction		0.719	0.992	0.915	0.738

^a–c^Means within a row column with no common superscripts differ significantly (*p* < 0.05). TP, total protein; ALB, albumin; TC, total cholesterol; GLU, glucose; Interaction, indicates the interaction effect of classical music and stocking density.

### 3.6. Meat quality

Breast muscle color and cooking loss of broilers were affected by different stocking densities (*p* < 0.05; Table 7). The L*_24h_ value of breast muscle in broilers in the 15.5 birds/m^2^ group was lower than that in the 20.3 birds/m^2^ group (*p* < 0.05), and the a*_24h_ value of breast muscle in broilers in 15.5 birds/m^2^ group was increased with the increase of stocking density. The cooking loss of breast muscle in broilers in 15.5 birds/m^2^ and 17.9 birds/m^2^ groups was lower than that in 20.3 birds/m^2^ groups (*p* < 0.05). With the increase in stocking density, the shear force of the breast muscle in broilers tended to increase (*p* = 0.059). Playing classical music increased the a*_24h_ value of breast muscle in broilers (*p* < 0.05). There was no interaction between classical music and stocking density on the meat quality of broilers (*p* > 0.05).

**Table 7:** Effect of classical music and stocking density on meat quality of broilers.

Items		Meat color			Cooking loss (%)	Shear force (N)
Stocking density (birds/m^2^)	Classical music	L*_24h_	a*_24h_	b*_24h_		
15.5	+	50.89 ± 1.70	7.43 ± 0.52	15.87 ± 1.30	10.89 ± 0.78	2.58 ± 0.12
	−	51.55 ± 4.51	7.15 ± 0.52	16.28 ± 1.06	11.06 ± 1.10	2.61 ± 0.15
17.9	+	52.85 ± 4.09	7.03 ± 0.47	16.65 ± 1.41	11.32 ± 1.02	2.70 ± 0.15
	−	55.30 ± 2.81	6.08 ± 0.49	15.91 ± 2.45	12.09 ± 0.89	2.60 ± 0.17
20.3	+	53.65 ± 4.45	5.81 ± 0.58	16.40 ± 1.22	12.64 ± 0.92	2.68 ± 0.15
	−	56.98 ± 4.66	5.86 ± 0.39	17.13 ± 1.59	13.06 ± 1.06	2.78 ± 0.13
Main factors						
Classical music	+	52.46 ± 3.61	6.52 ± 0.73	16.30 ± 1.28	11.61 ± 1.15	2.63 ± 0.14
	−	54.61 ± 4.49	6.16 ± 0.53	16.44 ± 1.77	12.07 ± 1.28	2.69 ± 0.16
Stocking density (birds/m^2^)	15.5	51.23 ± 3.27^b^	7.23 ± 0.52^a^	16.07 ± 1.15	10.97 ± 0.91^b^	2.59 ± 0.14
	17.9	54.07 ± 3.58^ab^	6.55 ± 0.67^b^	16.28 ± 1.94	11.70 ± 1.00^b^	2.64 ± 0.14
	20.3	55.31 ± 4.68^a^	5.83 ± 0.47^c^	16.76 ± 1.41	12.85 ± 0.97^a^	2.74 ± 0.14
*p*-value						
Classical music		0.106	0.024	0.797	0.167	0.245
Stocking density		0.041	<0.001	0.548	<0.001	0.059
Interaction		0.693	0.058	0.487	0.745	0.856

^a–c^Means within a row column with no common superscripts differ significantly (*p* < 0.05). L*, lightness; a*, redness; b*, yellowness; Interaction, indicates the interaction effect of classical music and stocking density.

## 4. Discussion

Under the high-stocking density, broilers become extremely sensitive to the environment and are susceptible to stress, which may lead to impaired growth and development. Mohammed et al. (20) reported that, compared with the stocking density of 14 and 18 birds/m^2^, the stocking density of 20 birds/m^2^ significantly reduced the ADFI, ADG, and F/G of broilers. Similar to the above results, this study showed that a stocking density of 20.3 birds/m^2^ significantly reduced broiler feed intake and body weight, and increased F/G and mortality. Stress induced by high-stocking density may be the main reason for the decreased growth performance of broilers. Moreover, the high mortality of broilers in this experiment at two densities of 17.9 and 20.3 birds/m^2^ may be due to the high number of broilers in the cages, and severe competition for food among the flock, resulting in sudden death due to severe stress (21, 22). In this study, body weight gain and F/G of broilers were improved by playing classical music. Similar to this result, Gvaryahu et al. (23) found that music increased daily weight gain in broilers, and Tolun et al. (24) showed that music significantly improved the F/G of Ross 308 broilers. The musical environment improved the growth performance of broilers, probably due to the exposure of broilers to music starting at 1 day of age, which enabled them to adapt to the audible environment, thus reducing various stresses and fears during growth, reducing the corresponding energy expenditure and improving feed conversion. This is in agreement with Hoffman (25) and Gaioni et al. (26) who suggested that music may be beneficial in reducing fear in young chicks. On the other hand, the overall mortality rate of broiler chickens in the experimental group dropped by 44%, which may be attributed to classical music modulating the animals’ nervous systems through sound waves to relieve stress (27, 28), this may also contribute to the reduction of mortality in broilers. In this study, there was an interaction effect between classical music and stocking density on broiler F/G. At the same stocking density, the F/G of broilers in the 17.9 and 20.3 birds/m^2^ groups playing classical music was significantly lower than that of the group without music, and the F/G of both groups decreased to the level of low stocking density without music intervention. This suggests that classical music can reverse the growth inhibition caused by increased stocking density and improve broiler growth performance.

When birds experience stress, their HPA axis is activated, and the pituitary gland is stimulated to secrete ACTH and glucocorticoids synthesized by adrenal cortical cells (29). Puvadolpirod et al. (30) found that the injection of high doses of exogenous ACTH increased corticosterone levels in the blood of broilers and increased stress levels by establishing a physiological stress model. In our study, serum CORT and ACTH levels were significantly higher in broilers in the 20.3 birds/m^2^ group than in broilers in the 15.5 birds/m^2^ group. Another study also showed that serum CORT levels were significantly increased when the stocking density of broilers was increased from 30 to 40 kg/m^2^ (31). This means that broilers at high stocking densities are more susceptible to physiological stress. Many researchers have reported a positive effect of classical music on alleviating stress in animals (8). We found that playing classical music significantly reduced these two indicators in broiler serum at stocking densities of 17.9 and 20.3 birds/m^2^. And there was no significant difference in serum CORT and ACTH levels between 17.9 and 15.5 birds/m^2^ and between the 20.3 birds/m^2^ group (playing classical music) and the 17.9 birds/m^2^ group (no music). This suggests that classical music can be useful in alleviating the stress caused by increasing stocking density in broilers. Similarly, Dávila et al. (28) indicated that the auditory enrichment obtained from classical music can reduce stress in chicks. It may be that music can reduce the stress response in the HPA axis by inducing activity in the hippocampus of the brain (11, 12), therefore, the stress-relieving effect of classical music on broiler chicks may be related to the ability to alter neuronal activity within these brain structures, however, the exact mechanisms involved need further investigation.

Oxidative stress can lead to biological damage and a variety of physiological disorders that can reduce the growth performance of broilers (32). Important antioxidant enzymes such as GSH-PX and SOD play an important role in combating oxidative damage. MDA is a product of lipid peroxidation and its concentration reflects the state of oxidative stress (33). Simsek et al. (6) reported that overcrowded rearing environments led to elevated blood MDA levels and increased oxidative damage in broilers. Li et al. (5) suggested that stress-induced lipid peroxidation effects may be due to decreased antioxidant enzyme activity at high stocking densities (18 birds/m^2^). The above-mentioned reports were similar to our results, with increased serum MDA levels and decreased CAT and GSH-PX activities in the 20.3 birds/m^2^ group compared with the other two densities. It is possible that the crowded space led to increased fighting behavior in broilers, resulting in metabolic disturbances that contributed to high lipid peroxidation and reactive oxygen species production *in vivo*, increased oxidative damage and MDA production in broilers, and decreased antioxidant enzyme activities (34, 35). In addition, we found that serum MDA concentration decreased, while serum SOD and GSH-PX activities tended to increase in broiler chickens by playing classical music. Previous studies have shown that classical music can increase the growth rate of human cells and increase the level of GSH (36). This implied that classical music may reduce oxidative stress damage by increasing the antioxidant enzyme activity of the body. On the other hand, classical music stimulation can increase positive behavior, reduce aggressive behavior, and relieve stress and anxiety in livestock (9, 37). In this study, it was hypothesized that classical music may serve to alleviate lipid peroxidation damage by reducing stress levels and increasing antioxidant enzyme activity in broilers.

Serum biochemical indicators reflect the nutritional metabolism, stress, and health status of the body (38–41). Mohammed et al. (20) showed that serum TP and ALB levels in broilers were significantly reduced at a stocking density of 20 birds/m^2^. In this study, with the increase of stocking density, serum TP and ALB levels of broilers decreased, while serum TC and GLU levels increased. The decrease in serum protein levels suggested that high stocking densities had a negative impact on nutrient absorption and metabolic function in broilers. Elevated TC levels may be related to the fact that overproduction of CORT levels upregulated gene expression for cholesterol synthesis and uptake in broiler muscle, inducing the accumulation of cholesterol in muscle (42). In addition, stress-induced increases in glucocorticoid levels promote the breakdown of proteins and fats in the body, providing raw material for gluconeogenesis, and resulting in increased GLU levels in the blood (43). In this study, serum GLU levels in broilers were significantly reduced after stimulation by classical music. Similar to the results of this trial, Tabrizi et al. (44) showed that playing music not only reduced blood GLU levels in patients after surgery but also maintained relatively stable cortisol levels compared to the control group (no interference). Finn et al. (45) also reported that listening to music reduced cortisol and blood GLU levels in humans. In our study, we hypothesized that classical music may reduce the secretion of CORT and ACTH in broilers by alleviating the stress response, thus lowering blood GLU.

The relative development of the thymus, bursa, and spleen can be used to assess the immune function and health status of poultry (46). Simitzis et al. (47) found that increased stress levels in broilers at high stocking density resulted in lower bursal weights. Sanchez et al. (48) showed that reduced lymphoid organ weight in poultry was associated with stress-induced high serum corticosterone levels. In this study, the spleen and bursa weight of broilers decreased with increasing stocking density. Elevated concentrations of corticosterone cause degeneration of the lymphoid organs spleen and bursa phalloides and dysregulate the immune response by depleting lymphocytes in germ cells (49–51). Musical stimulation promotes T-cell proliferation in stressed mice (52). This is similar to our results that playing classical music increased the relative weight of the spleen and bursa of broilers. Serum immunoglobulins are important indicators of humoral immunity in animals and are a direct reflection of the immune function of the organism. Li et al. (5) showed that high-stocking density (18 birds/m^2^) significantly reduced serum IgG and IgA levels in broilers. Similarly, Hafez et al. (53) reported that serum IgA, IgG and IgM levels were significantly higher in the normal density group (10 birds/m^2^) than in the high-stocking density group (20 birds/m^2^). In the present study, increased stocking density significantly reduced serum IgA, IgM, and IgG levels in broilers, probably because high-stocking density induced stress increased glucocorticoid levels and inhibited the body’s immune function (54). Li et al. (10) reported that classical music increased serum IgG levels and improved cytokine levels in piglets. We found that classical music increased the serum IgA and IgG levels in broilers. Classical music may affect immune function by stimulating the nervous system of broiler chickens and the autonomic signals emanating from it interact with immune cells to control their function and response (55). However, the exact mechanism is unclear and further research is needed. The results of this study showed that there was an interactive effect between classical music and stocking density on the serum IgG content of broilers, and the IgG content of the 17.9 birds/m^2^ group recovered to the same level as that of the 15.5 birds/m^2^ group after classical music intervention, which indicated that classical music could alleviate the immunosuppression triggered by increased stocking density to some extent.

The quality of the muscle is usually assessed by color, tethering power, and shear force (56). Wu et al. (57) showed that 24 h after slaughter, high-stocking density (18.6 birds/m^2^) significantly increased L* and decreased a* values of breast muscle in broilers compared to low stocking density (12.9 birds/m^2^) and also resulted in higher drip losses. Hosseini et al. (58) reported that water loss of breast muscle in broilers was significantly lower in the high-stocking density group (20 birds/m^2^) than in the stocking density group (10 birds/m^2^). Similar to these results, in this study, it was also found that L*_24 h_ value and Cooking loss of breast muscle in broiler increased with the increase of stocking density, whereas a*_24 h_ value of breast muscle decreased. It has been reported that the high brightness values of chicken meat may be related to oxidative stress caused by high-stocking density (59). The strength of muscle oxidative capacity is related to high red values (57). High-stocking density caused high levels of nitric oxide production in broilers, increasing the accumulation of reactive oxygen and reactive nitrogen in the muscle, leading to massive oxidation of proteins, which reduced the strength of the myogenic fiber gel and increased the Cooking loss of chicken meat (60). Therefore, the high stocking density in this experiment led to a decrease in meat quality by inducing oxidative stress in broilers, while playing classical music enhanced the antioxidant capacity of broilers and improved the breast muscle a*_24h_ values.

## 5. Conclusion

Our results suggested that high-stocking density (20.3 birds/m^2^) can reduce growth performance, induce a stress response, decrease antioxidant and immune function, and reduce the meat quality of broilers. However classical music stimulus can indeed alleviate the negative effects of high-stocking density on broilers to a certain extent. Therefore, this was an effective measure to relieve the stressful conditions and increase the growth performance and immune status of broilers raised at the high stocking density condition.

## Data availability statement

The original contributions presented in the study are included in the article/Supplementary material, further inquiries can be directed to the corresponding authors. The tracks used in this study are searchable on Himalayan Audio Platform (https://www.ximalaya.com/).

## Ethics statement

The animal study was reviewed and approved by the protocols for the animal study were approved by the Institutional Animal Care and Use Committee of Hebei Agricultural University and carried out under the Guidelines for the Care and Use of Laboratory Animals of China.

## Author contributions

XG and JG performed the experiments and drafted the manuscript. XG, ZF, and JG carried out the statistical analysis. BY, YL, HX, YH, JJ, and LL helped the revision of this manuscript. ZF and LL contributed to the supervision and guidance of the present study. All authors contributed to the article and approved the submitted version.

## Funding

This study was supported by the Hebei Provincial Key R&D Project (20326617D, Shijiazhuang, China) and the Hebei Modern Agricultural Industry Technology System Laying Hens & Brolier Innovation Team Construction Project (HBCT2018150203, Shijiazhuang, China).

## Conflict of interest

JJ was employed by Hebei Jiuxing Agriculture and Animal Husbandry Development Co., Ltd.

The remaining authors declare that the research was conducted in the absence of any commercial or financial relationships that could be construed as a potential conflict of interest.

## Publisher’s note

All claims expressed in this article are solely those of the authors and do not necessarily represent those of their affiliated organizations, or those of the publisher, the editors and the reviewers. Any product that may be evaluated in this article, or claim that may be made by its manufacturer, is not guaranteed or endorsed by the publisher.

## Footnotes

^1^ https://www.ximalaya.com/

## Abbreviations

CORT: corticosterone
ACTH: adrenocorticotropin
CK: creatine kinase
IgA: immunoglobulin A
IgG: immunoglobulin G
IgM: immunoglobulin M
T-AOC: total antioxidant capacity
CAT: catalase
SOD: superoxide dismutase
MDA: malondialdehyde
GSH-PX: glutathione peroxidase
TP: total protein
ALB: albumin
TC: total cholesterol
GLU: glucose
